# Neutralizing Effects of *Mimosa tenuiflora* Extracts against Inflammation Caused by *Tityus serrulatus* Scorpion Venom

**DOI:** 10.1155/2014/378235

**Published:** 2014-06-11

**Authors:** Mariana Angélica Oliveira Bitencourt, Maira Conceição Jerônimo de Souza Lima, Manoela Torres-Rêgo, Júlia Morais Fernandes, Arnóbio Antônio da Silva-Júnior, Denise Vilarinho Tambourgi, Silvana Maria Zucolotto, Matheus de Freitas Fernandes-Pedrosa

**Affiliations:** ^1^Laboratory of Pharmaceutical Technology and Biotechnology, Department of Pharmacy, Federal University of Rio Grande do Norte, Avenue General Gustavo Cordeiro de Farias, Petrópolis, 59012-570 Natal, RN, Brazil; ^2^Laboratory of Pharmacognosy, Department of Pharmacy, Federal University of Rio Grande do Norte, Natal, RN, Brazil; ^3^Laboratory of Immunochemistry, Butantan Institute, São Paulo, SP, Brazil

## Abstract

Scorpion bite represents a significant and serious public health problem in certain regions of Brazil, as well as in other parts of the world. Inflammatory mediators are thought to be involved in the systemic and local immune response induced by *Tityus serrulatus* scorpion envenomation. The aim of this study was to evaluate the effect of extracts of *Mimosa tenuiflora* on model envenomation. In mice, the envenomation model is induced by *Tityus serrulatus* venom. Previous treatment of mice with fractions from *M. tenuiflora* was able to suppress the cell migration to the peritoneal cavity. The treatment of mice with *M. tenuiflora* extracts also decreased the levels of IL-6, IL-12, and IL-1*β*. We concluded that the administration of the extract and fractions resulted in a reduction in cell migration and showed a reduction in the level of proinflammatory cytokines. This study demonstrates, for the first time, the anti-inflammatory effect of aqueous extract from the *Mimosa tenuiflora* plant on *T. serrulatus* venom.

## 1. Introduction

Scorpion bite represents a significant and serious public health problem in certain regions of Brazil, as well as in other parts of the world, due to the frequency of their occurrence and to their potential for inducing severe, even fatal, clinical manifestations, especially among children [1]. In Brazil, most fatalities result from bites received from the* Tityus serrulatus* scorpion. The Brazilian Ministry of Health reports approximately 8000 scorpion bites/year, and the mortality rate among children is 1% [2].

The specific signs of scorpion envenomation are directly related to the venom components, with some patients developing an inflammatory response. Although the production of pro- and anti-inflammatory cytokines in response to tissue injury is essential to repair tissue structure and function, excessive generation of proinflammatory cytokines can aggravate tissue damage [3]. Many different cytokines are released following severe envenomation. Increased interleukin IL-6 levels have been observed in plasma from patients with different grades of* T. serrulatus* envenomation. High levels of IL-6 and IL-1 were also observed in mice exposed to* Centruroides noxius* and* T. serrulatus* scorpion venoms [4–6]. Injection of scorpion venom into experimental animals produces systemic effects, with signs and symptoms similar to those observed in human envenomation, being fever, psychomotor agitation, salivation, lachrymation, increased gastrointestinal tract mobility, cardiac and respiratory arrhythmias, and arterial hypertension followed by hypotension, cardiac failure, pulmonary edema, and shock, among others [2, 7, 8]. These features have been traditionally explained by the effects of neurotransmitters released by the scorpion venom, but the release of mediators from the systemic inflammatory response syndrome may also play an important role [9].

The recommended treatment for envenomation is the intravenous administration of antivenom according to the severity of envenoming. The serum therapy is indicated in neutralizing venoms inoculated after incidence with venomous animals [10–12]. The serum has some disadvantages, having a series of adverse effects on the victim, such as anaphylaxis and hypersensitivity to heterologous proteins from serum and inefficiency in combating the local effects of the venom [13]. For these reasons, several studies have been conducted in the search for natural compounds that can complement the currently available serotherapy.

*Mimosa tenuiflora* (Willd.) Poiret (Leguminosae) is a tree popularly known in Brazil as jurema-preta [14]. The plant is distributed in areas of tropical deciduous forests in the Americas, from the southeastern regions of Mexico to northern Brazil and Venezuela, growing as secondary opportunistic vegetation. According to Brazilian and Mexican ethnopharmacological sources, the bark of this plant—once dried, powdered, and directly applied to the lesion—is an effective remedy for treating skin burns and wounds and preventing inflammation [15–18].

Basic preclinical studies report that aqueous and alcoholic extracts from dried* M. tenuiflora* bark are particularly rich in tannins, compounds which seem to play an important role in the healing mechanism; they possess* in vitro* antimicrobial properties against a broad group of Gram-positive and -negative microorganisms, yeasts, and dermatophytes [19, 20]. Also with treatment for severe skin ulcer, the positive effects of* M. tenuiflora* extracts were clearly related to the high content of polyphenols with a high cicatrization potential [18]. Other studies allowed identification of a group of triterpenoidal saponins from the bark, designated as mimonosides A–C [21], which, according to* in vitro* observations, induced cultured human-cell proliferation, possessed immunomodulation capacity, and, therefore, were attributed to at least part of the potential cicatrizing properties of the plant's bark [22, 23].

The aim of this study is to evaluate the neutralizing capacity of the extract of* Mimosa tenuiflora* on the inflammation induced by* Tityus serrulatus* scorpion venom.

## 2. Materials and Methods

### 2.1. Extraction

The bark of* Mimosa tenuiflora* was collected in the rural region of “Queimadas Mountain” (7°03_52_S/34°49_51_W), Parelhas, Rio Grande do Norte State, Brazil, in April 2011. The specimen was identified by Allan A. Roque. Voucher specimens of* M. tenuiflora* (JPB 13985) have been deposited in the Herbarium UFRN at the Universidade Federal do Rio Grande do Norte, Brazil.

### 2.2. Isolation

The bark of* M. tenuiflora* (500 g) was air-dried at room temperature, powdered, and extracted using hot water (100°C) by decoction (plant: solvent, 1 : 10, w/v) for 10 min. Then, the extracts were filtered through Whatman paper no. 1 and lyophilized.

Thereafter, the aqueous extract was resuspended in distilled water and partitioned with the following solvents: dichloromethane (3 × 300 mL), ethyl acetate (3 × 300 mL), and *n*-butanol (3 × 300 mL), yielding 750 mg of dichloromethane (CH_2_Cl_2_), 1.0 g of ethyl acetate (EtOAc), and 2.5 g of *n*-butanol (*n*-BuOH) fractions. The CH_2_Cl_2_, EtOAc, and *n*-BuOH fractions were analyzed by TLC using aluminum sheets of silica gel F254 (Merck). All chromatograms were developed in a saturated chamber. The following solvent system was used: ethyl acetate : acetic acid : formic acid : water (8 : 0.5 : 0.5 : 0.5 v/v/v/v). After the chromatograms were developed, the plates were dried and the spots were visualized sequentially under UV light at 254 and 365 nm. The plates were then sprayed with a methanol solution of sulfuric vanillin (4%) and Natural Product Reagent A 0.1% (NP-Reagent). Production and purification of extracts endowed with antivenom activity are patented processes (patent number PI033120000116, INPI).

### 2.3. Animals

Male BALB/c mice (6–8 weeks old) were used in the experiment. All mice were housed, 5-6 per cage, at a room temperature of 22 ± 2°C and a 12 h : 12 h light/dark cycle. They had free access to food and water. Groups of five animals were used in each test group and control animals received saline only. All* in vivo* experiments were approved by “Ethics Committee on Animal Use, CEUA/UFRN,” under protocol number 008/2011, which was in accordance with the guidelines of the Brazilian Committee for Animal Experimentation (COBEA).

### 2.4. Venom

Lyophilized* Tityus serrulatus* scorpion venom was kindly supplied by the Butantan Institute, São Paulo, SP, Brazil. The venom was prepared in PBS at 1 mg/mL concentration and was stored at −20°C until used.

### 2.5. Evaluation of* T. serrulatus* Venom-Induced Envenomation

In order to evaluate the envenomation induced by* T. serrulatus* and establish the challenge dose, groups of six male mice were injected intraperitoneally (i.p.) with 0.1, 0.2, 0.3, 0.4, and 0.8 mg/kg of* T. serrulatus* venom dissolved in isotonic saline. At the selected time points (4, 6, and 8 hours), the animals were anesthetized with ketamine/xylazine (80/10 mg/kg i.p.) and sacrificed by cervical dislocation, and peritoneal exudates were harvested by peritoneal wash with 3 mL of cold PBS. Exudates were centrifuged at 250 ×g for 10 min, at 4°C, and the total cell numbers were determined in a Neubauer chamber following staining with Turk's solution. After selecting the appropriate dose of venom and the time, the neutralization assay of venoming induced by* Tityus serrulatus* venom was performed by extracts of* M. tenuiflora.*

### 2.6. Assessment of Antivenom Activity

The envenomation was induced according to the procedure described previously in Pessini, 2006, with slight modifications. Animals were inoculated intravenously (i.v.) with saline, aqueous extracts from* M. tenuiflora* (20, 30, or 40 mg/kg), CH_2_Cl_2_, EtOAc, and BuOH fractions (40 mg/kg) and, five minutes later, the animals were injected intraperitoneally with sterile PBS or* T. serrulatus* venom (VTs) freshly prepared (0.1 mL of 0.8 mg/kg) in sterile PBS. According to the observed results, AcOEt fraction showed the best inhibition profile of inflammatory cells; therefore a dose curve response to EtOAc fraction (20, 30, or 40 mg/kg) was evaluated. After 6 h, the animals were anesthetized with ketamine/xylazine (80/10 mg/kg i.p.) and sacrificed by cervical dislocation, and peritoneal exudates were harvested by peritoneal wash with 3 mL of cold PBS. Exudates were centrifuged at 250 ×g for 10 min, at 4°C, and the total cell numbers were determined in a Neubauer chamber following staining with Turk's solution. Results were expressed as number of neutrophils/cavity. The supernatants were collected for determination of IL-6, IL-12, and IL-1*β* (pg/mL) levels, which was performed using an enzyme-linked immunosorbent assay kit from eBioscience (San Diego, CA, USA).

### 2.7. Statistical Analyses

Data are expressed as the mean ± standard deviation. Statistical analyses were performed by ANOVA and Tukey test and the level of significance was set at *P* < 0.0001.

## 3. Results

**Figure 1:**
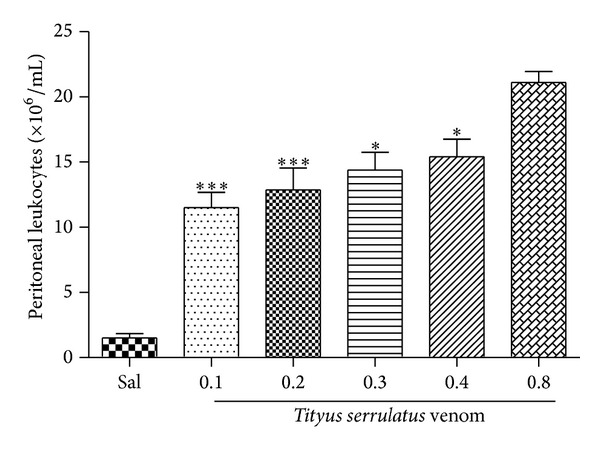
*Tityus serrulatus* venom-induced inflammation. BALB/c mice were injected i.p. with 0.1 mL of VTs (0.1, 0.2, 0.3, 0.4, or 0.8 mg/kg). After 6 hours, peritoneal lavage was performed with PBS and the cell number was determined in a Neubauer chamber. *N* = 6 , ***P ≤ 0.001, compared to PBS and 0.8 mg/kg group.

**Figure 2:**
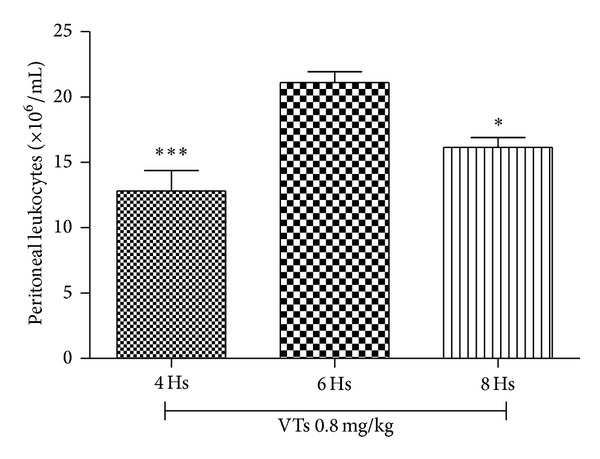
*Tityus serrulatus* venom-induced inflammation. BALB/c mice were injected i.p. with 0.1 mL of VTs (0.8 mg/kg). After 4, 6, and 8 hours, peritoneal lavage was performed with PBS and the cell number was determined in a Neubauer chamber. *N* = 6 , ***P ≤ 0.001, compared to PBS and 6-hour group.

The envenomation induced by VTs was assessed using an animal model of peritonitis. A significant increase in cell migration to peritoneal cavity was observed upon intraperitoneal injection of VTs. Among the doses tested, the strongest effect was obtained with 0.8 mg/kg (Figure 1). This dose was chosen as the challenge dose for evaluation of the antivenom activity of* M. tenuiflora* extract (after evaluating the effect of three different doses of* T. serrulatus*-induced envenomation). After selecting the dose, the kinetics (4, 6, and 8 h) of the venom-induced peritoneal cell migration was evaluated. The results presented in Figure 2 show that VTs induced a marked increase in the peritoneal cell migration at the three times analyzed (4, 6, and 8 h). The highest influx of cells in the peritoneal cavity was observed at 6 h. This time was, therefore, selected for the neutralization assay, where the antivenom activity of* M. tenuiflora* extract against venoming induced by* T. serrulatus* was evaluated.

**Figure 3:**
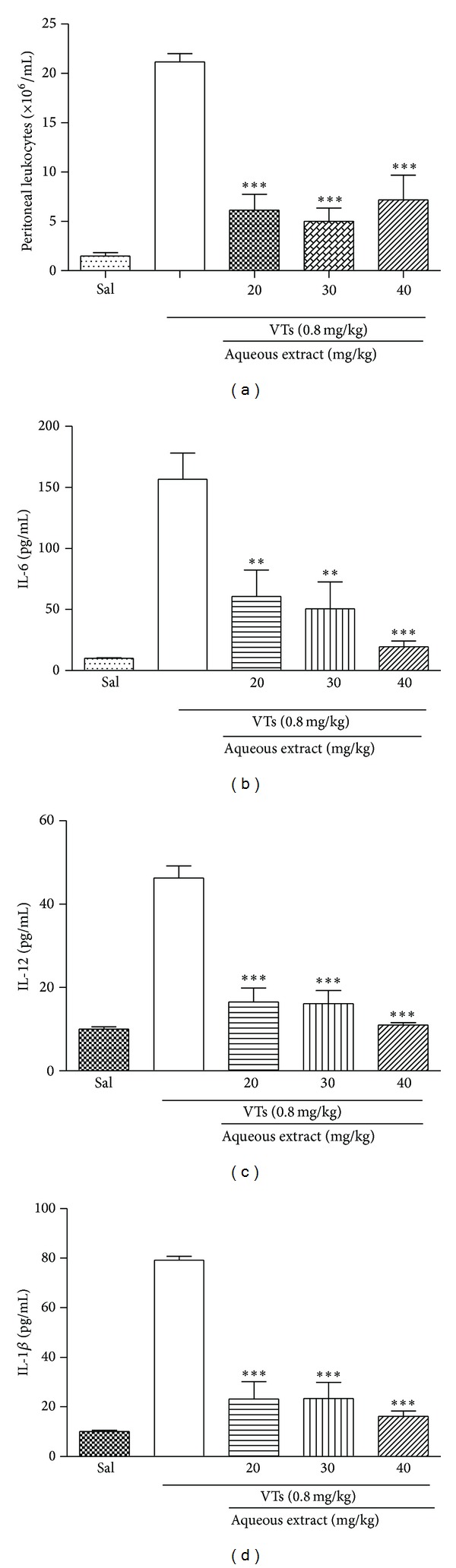
Effect of aqueous extracts of* M. tenuiflora* against* T. serrulatus* venom-induced inflammation. BALB/c mice were treated intravenously (i.v.) with PBS or aqueous extract at doses of 20, 30, and 40 mg/kg and, a few minutes later, were injected i.p. with 0.1 mL of VTs (0.8 mg/kg). After six hours, peritoneal lavage was performed with PBS and the cell number was determined in a Neubauer chamber (a). The supernatants were collected for determination of IL-6 (b), IL-12 (c), and IL-1*β* (d) levels, which was performed using an enzyme-linked immunosorbent assay. *N* = 6, *P ≤ 0.005, compared to PBS and drug-treated groups.

The effect of aqueous extract of* M. tenuiflora* was evaluated in a* T. serrulatus* venom-induced envenomation model. As expected, the animals treated intravenously (i.v.) with saline and 5 min later intraperitoneally (i.p.) with VTs showed intense leukocyte migration to the peritoneal cavity (Figure 3(a)). On the other hand, groups treated with three different doses (20, 30, or 40 mg/kg) of* M. tenuiflora* aqueous extract showed a significant inhibition of cell migration to the peritoneal cavity, as well as a reduction in the levels of IL-6 (Figure 3(b)), IL-12 (Figure 3(c)), and IL-1*β* (Figure 3(d)), when compared with the group that received saline i.v. and VTs i.p.  Table 1 summarizes the anti-inflammatory activity of all plant extracts tested regarding the inhibition of peritoneal cell migration, which was similar for all three doses of aqueous extract analyzed.

**Figure 4:**
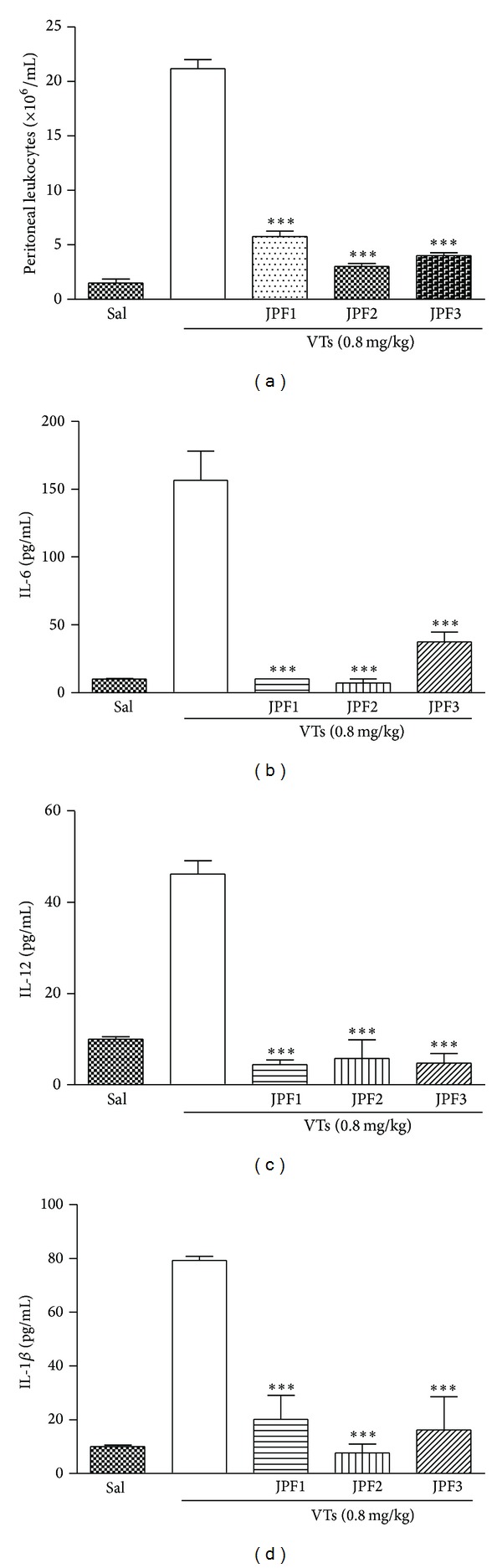
Effect of dichloromethane (CH_2_Cl_2_), ethyl acetate (EtOAc), and *n*-butanol (*n*-BuOH) fractions of* M. tenuiflora* against* T. serrulatus* venom-induced inflammation. BALB/c mice were treated intravenously (i.v.) with CH_2_Cl_2_ (JPF1), EtOAc (JPF2), and *n*-BuOH (JPF3) fractions (a) at a dose of 40 mg/kg and a few minutes later were injected i.p. with 0.1 mL of VTs (0.8 mg/kg). After six hours, peritoneal lavage was performed with PBS and the cell number was determined in a Neubauer chamber. The supernatants were collected for determination of IL-6 (b), IL-12 (c), and IL-1*β* (d) levels, which was performed using an enzyme-linked immunosorbent assay. *N* = 6 , *P ≤ 0.005, compared to PBS and drug-treated groups.

**Figure 5:**
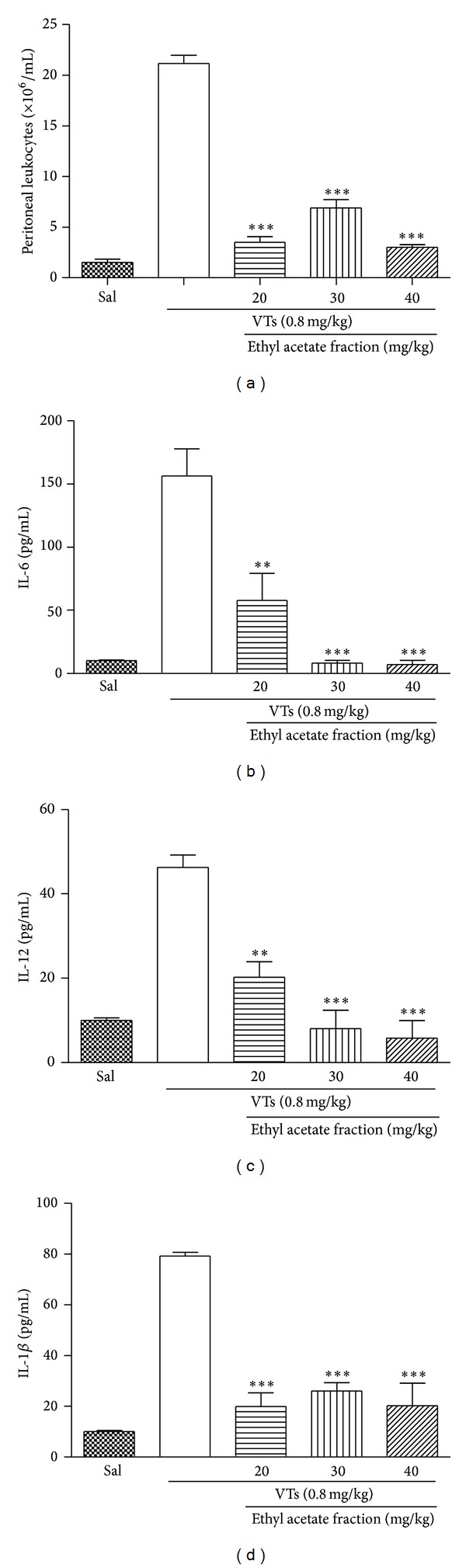
Effect of ethyl acetate fraction of* M. tenuiflora* against* T. serrulatus* venom-induced inflammation. BALB/c mice were treated intravenously (i.v.) with EtOAc fractions (a) at doses of 20, 30, and 40 mg/kg and a few minutes later were injected i.p. with 0.1 mL of VTs (0.8 mg/kg). After six hours, peritoneal lavage was performed with PBS and the cell number was determined in a Neubauer chamber. The supernatants were collected for determination of IL-6 (b), IL-12 (c), and IL-1*β* (d) levels, which was performed using an enzyme-linked immunosorbent assay. *N* = 6 , *P ≤ 0.005, compared to PBS and drug-treated groups.

Through phytochemical analysis of aqueous extract by TLC, a yellow spot (*Rf* 0.36/UV 365 nm) was observed after spraying the plate with NP-Reagent, suggesting the presence of flavonoids. Furthermore, when the plate was revealed with vanillin sulfuric, three red spots (*Rf* *s* 0.24, 0.34, and 0.43) and a yellow spot (*Rf* 0.36) were observed. According to literature data, these results suggest the presence of terpenes and/or steroids. Following a bioassay-guided fractionation process, the aqueous extract was partitioned with CH_2_Cl_2_, EtOAc, and *n*-BuOH. Each fraction was submitted to a screening in the same experimental model at the dose of 40 mg/kg. All groups treated with the fractions showed significant inhibition of cell migration to the peritoneal cavity (Figure 4(a) and Table 1). The CH_2_Cl_2_, EtOAc, and *n*-BuOH fractions showed a reduction in IL-6 (Figure 4(b)), IL-12 (Figure 4(c)), and IL-1*β* (Figure 4(d)) levels, respectively, when compared with the group administered with saline i.v. and VTs i.p. A dose-response profile (20, 30, and 40 mg/kg) was made for the EtOAc fraction (Figure 5(a)) which displayed a higher antivenom effect than other fractions. The effect on cell migration to the peritoneal cavity was not dose-dependent, since the three doses tested induced similar inhibition. On the other hand, the ability of EtOAc fraction to reduce the production of IL-6 (Figure 5(b)), IL-12 (Figure 5(c)), and IL-1*β* (Figure 5(d)) was dose-dependent. A study of toxicity was also performed “*in vitro*” using cell T3T, where the aqueous extract showed dose-dependent toxic effect but only at very high doses (data not shown).

## 4. Discussion

In this study, we have examined the antivenom effect of* M. tenuiflora* extract and fractions on inflammation caused during the envenomation induced by* T. serrulatus* venom. Scorpion venom consists of a mixture of neurotoxins that interact with Na^+^, K^+^, Ca^+^, and Cl^−^ channels, depending on their pharmacological properties and their physical interaction with the channels. Toxins that act on voltage-gated ion channels play a role in the immune response and trigger the release of inflammatory mediators [6]. Severe envenomation induced in mice injected with VTs was associated with an elevation in the serum levels of various cytokines and in the early stages of the acute-phase response, where neutrophils were the predominant blood cells, due to the increased white cell counts being a common finding in scorpion envenomation [24, 25]. Mobilization of either marginated cells or bone marrow reserve can increase blood neutrophil numbers rapidly due to the action of catecholamines, which are known to be released by scorpion venom and induce leukocytosis through mobilization of the marginated pool [26]. It is also probable that the hematopoietic stimulation effects of IL-1 and IL-6 also play a role [25], as they are also platelet-activating factor (PAF) receptor-dependent [27]. The proinflammatory cytokines induce local and systemic inflammatory manifestations. The local effects include the activation of vascular endothelium, an increase in vascular permeability, and access of leukocytes to the affected tissue and their activation and local tissue destruction [28].

We have found that intraperitoneal injection of VTs was able to induce an inflammatory reaction, which is revealed by high levels of IL-1*β*, IL-6, and IL-12 and increased cell migration to the peritoneal cavity. In the present study, we demonstrated that the groups treated with aqueous extract and fractions from* M. tenuiflora* showed a significant inhibition of cell migration to the peritoneal cavity and also showed a reduction in the levels of IL-6, IL-12, and IL-1*β*. The extract-treated groups exhibited antivenom activity, revealing impairment of leukocyte migration.

There are, to our knowledge, no previous studies in the literature presenting antivenom activity of extracts from* M. tenuiflora*; however, it is known that there is a significant presence of saponins in this species [18, 21, 29]. One hypothesis for this activity is the inhibition of the production of leukotrienes and prostaglandin E2 by inhibiting the enzyme phospholipase A2 and COX2, respectively, as well as blocking the release of all other inflammatory mediators involved in the venoming, as studies being conducted in parallel to our laboratory previously demonstrated the anti-inflammatory potential of* M. tenuiflora* in experimental models of inflammation using carrageenan. Regarding envenomation-induced inflammation as studied in the present work, it is known that the scorpion venoms can stimulate the immune-neuroendocrine axis by inducing the release of catecholamines, corticosteroids, bradykinin [30–33], and eicosanoids mediators, such as prostaglandin (PG)E2, lipoxin A2 (LXA2), and leukotriene (LT)B4 [2, 34], which are derived from the enzymatic oxygenation of arachidonic acid (AA). These signal molecules control key cellular processes, including cell activation, metabolism, migration, cell proliferation, and death [35, 36].

Other studies in the literature have already demonstrated that PGE2 is involved in the inflammatory response and in the neutrophil recruitment [37] in mice inoculated with* T. serrulatus* scorpion venom [38]. As in certain sodium channel toxins and potassium (Ts2 and Ts6),* T. serrulatus* is involved in the release of cytokines and cell migration by inducing the production and release of PGE2 and LTB4 [39]. As already has been stated, the aqueous extract of* M. tenuiflora* is rich in saponins and tannins [18]. Some studies showed the presence of flavonoids and saponins which have been previously reported to have anti-inflammatory efforts* in vivo* and* in vitro*. For example, some saponins and flavonoids were reported to suppress the release of proinflammatory mediator production by inflammatory agents in macrophages [40–43]. Some studies have shown that the mechanism of saponins and flavonoids in anti-inflammatory activity may be mediated by inhibiting the activation of nuclear factor-kB, thus resulting in decreased expression of NF-kB-regulated proteins such as inducible nitric oxide synthase (iNOS) [44–46]. Other reports revealed that tannins have the ability to bind to proteins [47, 48], suggesting that tannins could inactivate venom toxins preventing their toxic activity. As envenomation by* Tityus serrulatus* triggers an intense inflammatory response, it is possible that the anti-inflammatory potential of active components in* M. tenuiflora* extract, as well as other possible biological activities, contributes to the antivenom activity of the plant.

## 5. Conclusion

In conclusion, this is the first study to evaluate the anti-inflammatory activity of extracts of* Mimosa tenuiflora* using scorpion venom. We demonstrated that the aqueous extract and fractions from* M. tenuiflora* exhibited important anti-inflammatory activity in a* T. serrulatus* venom-induced envenomation model. As expected, we have found that the animals that received intraperitoneal injection of VTs were able to induce an inflammatory reaction, which is revealed by high levels of IL-1*β*, IL-6, and IL-12 and increased cell migration to the peritoneal cavity. On the other hand, groups treated with fractions showed a significant inhibition of cell migration to the peritoneal cavity and showed a reduction in levels of IL-6, IL-12, and IL-1*β*. Further studies are required to determine the chemical composition of* M. tenuiflora* extract and its possible anti-inflammatory mechanisms of action.

## Figures and Tables

**Table 1:** Anti-inflammatory activity of *Mimosa tenuiflora* against* T. serrulatus *venom-induced envenomation.

Groups	Dose (mg/kg)	Cell migration	Inhibition (%)
Saline	—	21.17 ± 0.8292	
Aqueous extract	20	6.167 ± 1.600	71***
Aqueous extract	30	5.000 ± 1.366	76***
Aqueous extract	40	7.200 ± 2.498	66***
CH_2_Cl_2 _fraction	40	5.750 ± 0.5204	73***
*n*-BuOH fraction	40	4.000 ± 0.2887	81***
EtOAc fraction	20	3.500 ± 0.5774	83***
EtOAc fraction	30	6.900 ± 0.8124	67***
EtOAc fraction	40	3.000 ± 0.2887	86***

Values are mean ± standard deviation (S.D.), *n *= 6, ***P < 0.0001, compared to saline group.
